# The canine gut microbiome is associated with higher risk of gastric dilatation-volvulus and high risk genetic variants of the immune system

**DOI:** 10.1371/journal.pone.0197686

**Published:** 2018-06-11

**Authors:** Meredith A. J. Hullar, Johanna W. Lampe, Beverly J. Torok-Storb, Michael A. Harkey

**Affiliations:** 1 Public Health Sciences Division, Fred Hutchinson Cancer Research Center, Seattle, Washington, United States of America; 2 Department of Transplantation Biology, Clinical Research Division, Fred Hutchinson Cancer Research Center, Seattle, Washington, United States of America; University of Minnesota College of Veterinary Medicine, UNITED STATES

## Abstract

**Background:**

Large and giant dog breeds have a high risk for gastric dilatation-volvulus (GDV) which is an acute, life-threatening condition. Previous work by our group identified a strong risk of GDV linked to specific alleles in innate and adaptive immune genes. We hypothesize that variation in the genes of the immune system act through modulation of the gut microbiome, or through autoimmune mechanisms, or both, to predispose dogs to this condition. Here, we investigate whether differences in the canine fecal microbiome are associated with GDV and are linked to previously identified risk alleles.

**Methodology/Principle findings:**

Fecal samples from healthy Great Danes (n = 38), and dogs with at least one occurrence of GDV (n = 37) were collected and analyzed by paired-end sequencing of the 16S rRNA gene. Dietary intake and temperament were estimated from a study-specific dietary and temperament questionnaire. Dogs with GDV had significantly more diverse fecal microbiomes than healthy control dogs. Alpha diversity was significantly increased in dogs with GDV, as well as dogs with at least one risk allele for *DRB1* and *TRL5*. We found no significant association of dietary intake and GDV. Dogs with GDV showed a significant expansion of the rare lineage Actinobacteria (p = 0.004), as well as a significantly greater abundance of Firmicutes (p = 0.004) and a significantly lower abundance of Bacteroidetes (p<0.004). There was a significant difference in the abundance of 10 genera but after correction for multiple comparisons, none were significant. Bacterial phyla were significantly different between controls and dogs with GDV and at least one risk allele for *DRB1* and *TRL5*. Actinobacteria were significantly higher in dogs with GDV and with one risk allele for *DRB1* and *TLR5* but not *DLA88* genes. Furthermore, Collinsella was significantly increased in dogs with at least one risk allele for *DRB1* and *TLR5*. Logistic regression showed that a model which included Actinobacteria, at least one risk allele,and temperament, explained 29% of the variation in risk of GDV in Great Danes.

**Conclusions:**

The microbiome in GDV was altered by an expansion of a minor lineage and was associated with specific alleles of both innate and adaptive immunity genes. These associations are consistent with our hypothesis that immune genes may play a role in predisposition to GDV by altering the gut microbiome. Further research will be required to directly test the causal relationships of immune genes, the gut microbiome and GDV.

## Introduction

Large and giant dog breeds have a high risk for gastric dilatation-volvulus (GDV) [1–6] This life threatening condition involves the accumulation of gas in the stomach (dilatation) primarily from bacterial fermentation in the stomach [7]. Torsion of the stomach on its axis (volvulus) results in increased pressure leading to compression of gastric, cardiac, and other blood vessels. We previously posed the hypothesis that variations in certain genes of the immune system may predispose dogs to GDV, through modulation of the gut microbiome [8]. Briefly, this hypothesis is based on four types of evidence, as described more fully below: 1) that GDV has been associated with inflammatory bowel disease (IBD) in dogs, 2) that IBD is associated with dysbiosis of the gut microbiome, 3) that specific variants of certain immune genes play a significant role in both microbiome dysbiosis and the etiology of IBD, and 4) that our previous work has established an association of specific immune gene alleles to GDV in Great Danes. [9–17] In contrast, others have posited that aerophagia (gulping of air) may increase risk of GDV, however, analysis of gas composition from dogs with GDV suggests that the gas is from fermentation and not atmospheric sources. Regardless, if left untreated, GDV can progress to tissue damage, shock, and death. Several risk factors have been identified including diet [18, 19] and feeding regimes [2], age [1, 4], behavior [2, 20], and genetics [1, 3–5] suggesting that both environmental and genetic factors influence the risk of GDV.

Previously, we identified variants of genes in both the adaptive and innate immune systems, toll-like receptor 5 (TLR5) and the dog version of human leukocyte antigen of the Major Histocompatibility Complex genes (DLA88; Class 1 and DRB1, Class 2), that were associated with increased risk of GDV [8]. Toll-like receptor 5 detects pathogen-associated molecular patterns (PAMPs) that recognize bacterial flagellin [21]. Mutations in the TLR-5 gene or bacterial modification of flagellin are associated with increased risk of inflammation-based diseases [13, 22–27]. DLA88 and DRB1 are Class I and Class II DLA genes. They encode an array of proteins that serve both to signal “self” to the immune system, to block auto-immune destruction, and to present microbial antigens to the immune system. Class I genes code proteins that signal cytotoxic T cells and Class II code genes that signal T helper cell proteins that regulate the expansion of antigen-specific response by B-and T-cell clones. In particular, Th17 produces IL-17 a cytokine that has been associated with microbial infections. Class I and II genes also play a role in regulating the exposure of the host to pathogenic bacteria and maintain homeostasis of the gut microbiome. Mutations in these genes can influence host health by failure to detect certain pathogens or by mounting an autoimmune response against host tissues and cells which are factors in autoimmune-based diseases such as rheumatoid arthritis or celiac disease [28–33]. In dogs, our recent work showed that mutations in these genes were associated with increased risk of GDV [8].

The healthy gut microbiome in dogs is complex, and like the human microbiome, a stable microbiome is critical to canine health [33, 34]. In dogs, the microbiome is affected by many factors including diet, age, breed, or environment [33–35]. Alpha diversity, which determines the species richness and evenness within the microbial community, and beta-diversity which determines the shared diversity between microbiota in terms of various ecologic distances are often used as indicators of canine health [33, 34]. In healthy dogs, the dominant phyla include Bacteroidetes, Firmicutes, Fusobacteria, and Proteobacteria although the proportions may vary in individual dogs [33, 34]. Actinobacteria represent a rare but ubiquitous lineage [28] and canines also have micro-organisms in non-bacterial domains of life including eukaryote, archaea, and viruses [34].

A microbiome that is out of balance (dysbiosis) may predispose dogs to increased risk of diseases associated with the immune system. Relevant to a dysregulated immune function in GDV, an altered abundance of Bacteroidetes, and Proteobacteria has been associated with irritable bowel disease [17] and other autoimmune-mediated diseases in dogs [36]. Underlying mechanisms include 1) loosened tight junctions between gut epithelial cells which allow endotoxin to enter systemic circulation mounting an immune response through innate immune receptors, 2) mutations in immune genes that reduce detection of pathogens [37, 38], 3) modification of flagellin by bacteria to evade host surveillance [39], and 4) shifts in the microbial community that enrich for microbial metabolites associated with increased inflammation [35, 40]. GDV is reported to co-occur in dogs with inflammatory bowel disease [9], another inflammation-based condition with an auto-immune component, and a link to microbial dysbiosis [41]. Here, we address the hypothesis that variation in the genes of the immune system act through modulation of the gut microbiome, or through autoimmune mechanisms, or both, to predispose dogs to this condition. We hypothesize that the diversity and composition of the gut microbiome in dogs with higher risk of GDV are different than that in dogs without GDV, and that these differences are associated with the genetic variants previously linked to GDV [8].

## Materials and methods

### Study animals

#### Animal welfare statement

Owner consent was obtained for all participating dogs. All procedures, authorization forms, information packets and questionnaires used in this study were submitted to, and approved by the Institutional Animal Care and Use Committee (IACUC #50836) at Fred Hutchinson Cancer Research Center.

#### Selection of study animals

The purpose of this collection was to observe the steady-state profile of the gut microbiome in these dogs. In order to minimize the potential effects of the GDV event itself, or of the subsequent surgery and/or antibiotic treatments, we restricted stool sample collections to at least 3 months after any surgery, antibiotic treatments, or symptoms of gastric problems.[42] This restriction applied to both control and GDV dogs. Great Danes were recruited through an email network of breeders and owners. All interested owners were sent a questionnaire about their dog’s diet, exercise, temperament, coat color, medical history and family history of GDV. Two groups of Great Danes were selected, based on the presence or absence of GDV in their lifetime. Dogs chosen for the GDV group had at least one episode of GDV that required emergency intervention by a veterinarian. Dogs chosen for the control group had never experienced either severe gastric dilatation or torsion, nor had they experienced any other major gut-related problems. All dogs that had received prophylactic gastroplexy were excluded from the study as were all dogs that received antibiotics in the three months prior to stool collection. No attempt was made to restrict participation based on sex, diet, exercise level, coat color, or age. The owners of 178 Great Danes volunteered to enroll their dogs in the study. Of these, 80 dogs met the inclusion criteria for the study, including complete questionnaire data, confirmation of specific group criteria, and willingness to collect and send blood or buccal swab samples: 38 dogs met the criteria for the GDV group and 42 met the control group criteria. Samples were genotyped as previously described [8].

#### Diet

Dietary information was collected using a questionnaire that asked owners about feeding regime and diet content. If a commercial dog food was identified, we used the nutrient analysis given by the manufacturer and adjusted for daily total amount fed. If a non-commercial diet was fed, we estimated kcal and macronutrient content based on average values for red meat, poultry, and crude fiber, and adjusted for the total amount fed (Table 1). Data are presented as g/day.

**Table 1:** Dietary* and demographic characteristics for dogs with and without GDV. Data presented as mean (SD).

	Control**	GDV**	*P* **
**Protein, g/day**	173.3 (57.2)	186.5 (71.1)	ns
**Fat, g/day**	103.4 (36.5)	105.2 (45.6)	ns
**Carbohydrate, g/day**	301.7 (121.9)	276.0 (138.3)	ns
**Crude Fiber, g/day**	22.5 (8.2)	21.4 (11.2)	ns
**Energy, kcal/day**	2211.8 (746.2)	2157.5 (697.0)	ns
**Age, y**	4.5 (2.4)	6.6 (2.9)	0.036
**Age at GDV onset, y**		4.6 (2.9)	

*Dietary data are presented as g fed per day

**Logistic regression

### Stool samples

#### Collection

Stool samples were collected in RNAlater^TM^, frozen in home freezer, returned to the clinic, and then stored at −80°C, as previously described [43, 44].

#### DNA extraction and sequencing

DNA extraction and 16S rRNA gene sequencing methods have been previously described [43, 44]. Briefly, DNA was extracted from stool using physical and chemical lysis. The V1-V3 region of the 16S rRNA gene was amplified and sequenced using paired end MiSeq primers 27f and 519r (Illumina Mi-Seq; San Diego) 27F mod forward PCR primer sequence 5’-AGRGTTNGATCMTGGCTYAG-3’ and the 519R reverse PCR primer sequence 5’- GTNTTACNGCGGCKGCTG-3’.[45] following standard protocols (Molecular Research, Shallowater, TX).

#### Stool microbiome bioinformatics analysis

To classify sequences to bacterial taxonomy, sequences were processed using QIIME (v.1.8) [46]. Sequences were joined with the fastq-join method, using min_overlap = 15 and perc_max_diff = 12. Sequences were filtered with split_libraries_fastq.py with q parameter set to 25, and defaults otherwise. The Nelson two-step method was used for OTU generation using the SILVA database (release 111 [47–49], clustered at the 97% similarity level) in the closed reference OTU picking step. The OTU table was filtered using the QIIME script filter_otus_from_otu_table.py with—min_count_fraction set to 0.00005 as recommended in Navas-Molina et al. [50]. An additional filtering step set entries in the OTU table to zero if the number of observations was less than 10 per-sample, per-OTU. Additional OTU entries were filtered out if they were detected as chimeras using QIIME’s identify_chimeric_seqs.py script with method blast_fragments. Sequences were aligned to the Silva 16S rRNA gene reference alignment using the NAST algorithm [51]. The sequences were classified using MOTHUR’s naive Bayes classifier trained against the SILVA database (release 111, clustered at the 97% similarity level) [52]. Files were generated with number of sequences per sample at the phyla and genera taxonomic level classification and OTUs were used in subsequent statistical analysis. Following rarefaction, alpha diversity was assessed using OTU abundance for the Shannon diversity estimate, beta diversity was estimated using OTU abundance using the using weighted UNIFRAC and both estimates were used in statistical analysis [53].

#### Statistical analysis

Temperament, at least one risk allele for DLA88, DRB1, or TRL5, age at onset, and age were determined previously to be associated with increased risk of GDV and are included in the present analysis. In addition, we assessed whether diet was different between control and GDV dogs by sex using two sided t-tests (Table 1). Correlation analysis (Pearson’s) was used to assess associations between dietary intake, alpha diversity, and Phyla. For more details of other variables that were measured in the population see Harkey et al. [8].

Alpha diversity between control and GDV dogs and whether alpha diversity was associated with having at least one risk allele for TLR5, DLA88, or DRB1 was assessed by t-tests [54]. Global differences in the microbial communities between control and GDV dogs were assessed using MRPP on the UNIFRAC distance matrix [55–57].

Data are presented as the mean and standard deviation of untransformed relative percent of total sequencing counts per sample. Prior to statistical analysis, sequence counts of phyla and genera in each sample were normalized using centered log ratio [58]. All values had one added to the sequence count prior to calculating the centered log ratio. We assessed the abundance of phyla and genera in control and GDV dogs using Kruskal-Wallis nonparametric tests. Multiple comparisons were adjusted for using the Benjamini-Hochberg approach [59]. Backward stepwise logistic regression was used to model the effect of the microbiome (represented as the phyla Firmicutes, Bacteoridetes, and Actinobacteria), temperament, and at least one risk allele on risk of GDV. Five variables were added to the model with probability of entry (0.1) and removal (0.2).The performance of the model was assessed using an ROC curve.

## Results and discussion

### Characteristics

Of the 178 Great Dane owners that volunteered to include their dogs in this study, 83 dogs had stool samples collected. Of those stool samples, 8 did not pass our DNA QC criterion (i.e., low yield or poor quality) and were not sent for sequencing. Stool samples were obtained from 38 dogs in the control group and 37 in the GDV group. Of the 75 samples that were sequenced, we lacked genetic data for 8 dogs in the control group. Complete information about the family sets and SNP analysis were published previously [8]. We were able to obtain dietary information from 64 dogs, 34 control dogs and 30 GDV dogs (Table 1).

### Bioinformatic analysis

In total, we generated 2.3 million reads with an average of 25 K reads per sample and the average length of the joined reads was 371bp (±115). After bioinformatic processing, we identified 7 phyla, 51 genera and 464 OTUs in the stool samples. Sequence files are available in the Short Read Archive under submission number SUB3668349.

### Statistical analysis

We found that the GDV dogs were significantly older than the control dogs (p = 0.036) by about 2 years although our previous study showed that the control dogs had the same mean age as the GDV dogs at the time of GDV event. There were no significant differences in dietary intake between the two groups (Table 1).

**Fig 1:**
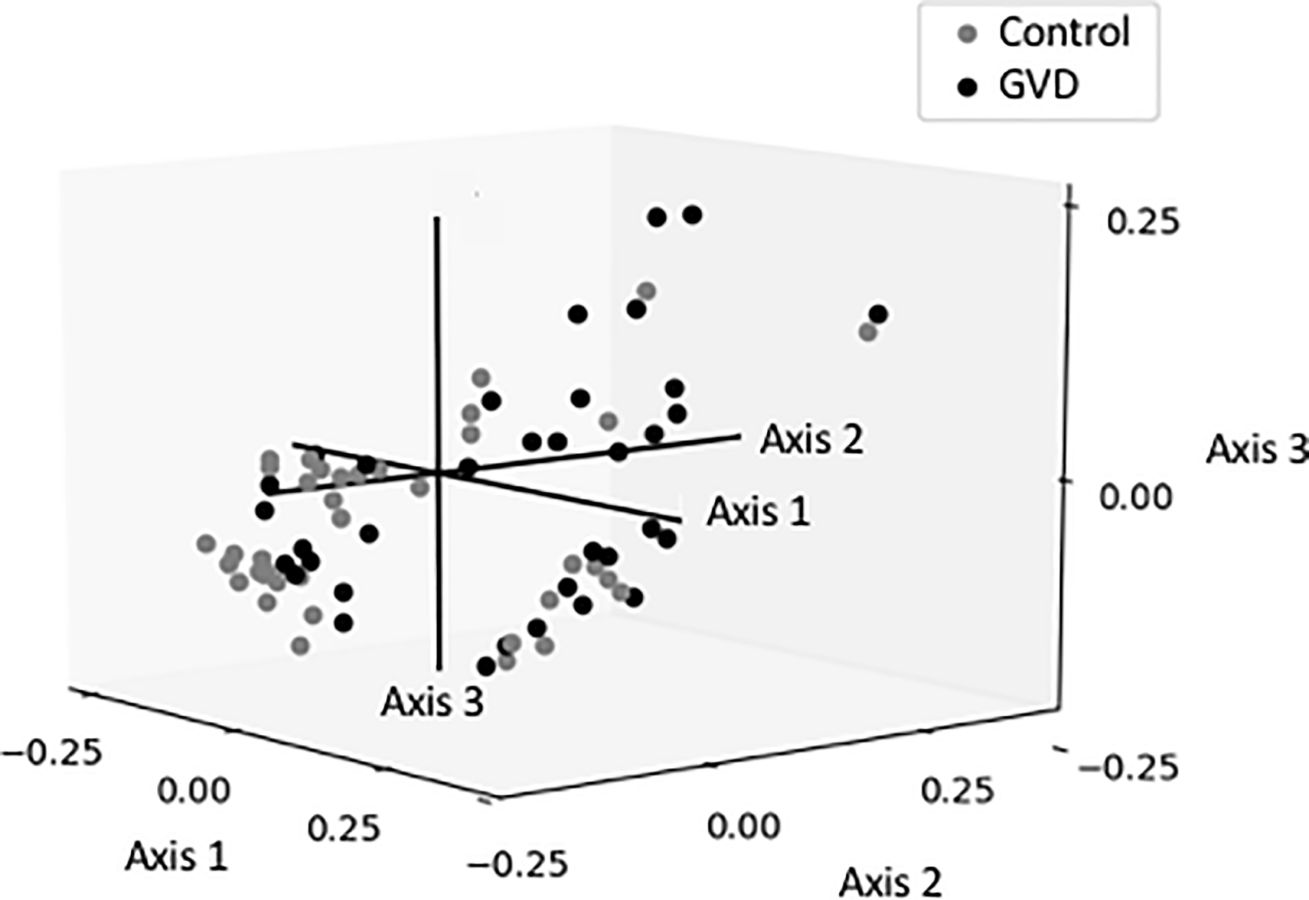
Multivariate analysis using a weighted Unifrac distance matrix of the gut microbiome in control (gray) and dogs with GDV (black).

Alpha and beta diversity were used to assess global differences in the microbiome in the GDV and control dogs. Alpha diversity determines the species richness and evenness within the microbiota while beta-diversity determines the shared diversity between microbiota in terms of various ecologic distances [54, 60]. Alpha diversity varied between the control and GDV dogs. There was a significantly higher Shannon diversity index in dogs with GDV (n = 37, 4.1 ±0.65) compared to control dogs (n = 38, 3.37 ±0.92; p = 0.002). In addition, there were significant differences in diversity between control and GDV dogs with at least one risk allele in TLR5 (4.29 ±0.55; p = 0.0005), DRB1 (4.13 ±0.49, p = 0.012), or DLA88 genes (3.90 ±0.89; p = 0.02). When beta diversity was assessed using the weighted UNIFRAC metric, it explained 26.6% of the variation in the overall microbiome composition in three axes. Axis 1 explained most of the variation (13.8%) (Fig 1), although there was no evident clustering of dogs by disease state.

In the canine microbiome, the phyla included Actinobacteria, Bacteroidetes, Firmicutes, Fusobacteria, Proteobacteria, Tenericutes, and Verrucomicrobia (Table 2). We found significant differences in the abundance of bacterial phyla between control and GDV dogs (Table 2). Bacteria in the phyla Actinobacteria (0.14% vs 0.49%, p = 0.004) and Firmicutes (25% vs 38%; p = 0.004) were significantly higher in dogs with GDV whereas Bacteroidetes were significantly lower (62% vs 47% (p = 0.004). In addition, there were differences in the phyla Actinobacteria (KW, p<0.06) and Bacteroidetes (KW, p<0.02) between control dogs and GDV dogs with at least one risk allele for DRB1, TLR5, or DLA88. More specifically, Actinobacteria were significantly higher in dogs with at least one risk allele in TLR5 (KW;p<0.02) and Firmicutes were significantly enriched in dogs with one risk allele in DRB1 (KW; p<0.012).

**Table 2:** Phyla (%) in cases vs controls.

	Controls* (n = 38)	GDV* (n = 37)	*P* **
**Bacteria;__Actinobacteria**	0.14 (0.34)	0.49 (0.66)	0.004
**Bacteria;__Bacteroidetes**	62.98 (21.95)	47.93 (22.41)	0.004
**Bacteria;__Firmicutes**	25.68 (17.46)	38.71 (20.27)	0.004
**Bacteria;__Fusobacteria**	7.80 (7.45)	6.56 (6.89)	0.341
**Bacteria;__Proteobacteria**	3.09 (4.61)	5.84 (9.30)	0.455
**Unknown;Other**	0.02 (0.08)	0.04 (0.08)	0.107

^*^ Mean (SD)

^**^ Non parametric t-test (Kruskal Walis) Benjamini Hochberg correction for multiple comparisons.

The broad phylogenetic trends were further reflected at the genera level (Table A in S1 Tables). We found differences in the abundance of 10 genera between control and GDV dogs. These genera were Collinsella, Prevotella, Lactobacillus, three members of the Clostridales, and three members of the Proteobacteria, including Helicobacteria and Escherichia/Shigella group. In the dogs with GDV, Collinsella, Lactobacillus, two member of the Peptostreptococcaceae, a member of the Escherichia_Shigella group in the Enterobacteriaceae, and another gamma-Proteobacteria were enriched in the microbiome. It is important to note that the phyla Actinobacteria only contained one genera, Collinsella. In contrast, Helicobacter and Prevotella were lower in the GDV group. However, upon correction for multiple comparisons (124 comparisons), they were no longer significant.

Backward stepwise logistic regression showed that a final model included Actinobacteria, having at least one risk allele of TLR5, DRB1, or DLA88, and nervous temperament explained 29% of the variation in risk of GDV (Tables A and B in S2 Tables). ROC curve analysis for the same variables resulted in an AUC of 0.86 suggesting that this model discriminates well between control and dogs at risk of GDV (S1 Fig).

## Discussion

Both environmental and genetic factors can influence disease outcomes that are linked to the composition of the microbiome and canine health [33–35]. In a previous analysis of this study population of Great Danes, we showed that variants of DLA88, DRB1, and TLR5 were associated with increased risk of GDV [8]. Differences in the canine microbiome have been associated with inflammation-based diseases, which are often linked to innate and adaptive immune deficiencies. We tested the hypothesis that the presence of at least one risk allele and the microbiome may influence the risk of GDV. In the present analysis, there was a significantly higher alpha diversity in dogs who previously had GDV. Differences in the composition of the microbiome and having at least one risk allele in TLR5, DLA88, and DRB1 were linked to increased risk of GDV.

The gut microbiome in healthy dogs has been characterized as diverse with abundant representation of bacterial, archaeal, and fungal microorganisms [33–35, 61]. In healthy dogs, after adjusting for diet, gut microbial communities vary with age, size, and breed [35]. We found that dietary intake was not associated with risk of GDV, although age was an important factor (Table 1). In our study, healthy dogs without GDV had a similar alpha diversity to that of healthy dogs in other breeds [33–35]. In the healthy, control Great Danes, Bacteroidetes and Firmicutes were the most abundant and prevalent phyla (Table 2). Fusobacteria and Proteobacteria also comprised between 3 and 8% of the canine microbiome. Actinobacteria were more rare (<1%). Studies of healthy dogs that showed that the three most abundant phyla were Bacteroidetes, Firmicutes, and Fusobacteria in other canine breeds [61, 62]. Low abundance of Actinobacteria has been reported in both healthy humans and dogs [28, 63].

Several lines of evidence suggest that there may be an interaction between diet and microbial diversity. We found a borderline significant inverse correlation with protein intake and alpha diversity. Different diets may cause the expansion of microbiota that specialize in the metabolism of particular dietary components and this can be captured in diversity measures [64]. Li et al found that dogs responded with increased diversity to a diet high in protein and low in carbohydrate [65]. Interestingly, others have found that a diet that included raw meat also increased the gut microbiome diversity which may reflect the structural complexity of the types of tissues in raw meat that need to be metabolized by the microbiome compared to processed kibble or canned food. In contrast, other studies that have shown prebiotics or complex carbohydrates in dog food can increase diversity [64].

Expansion of rare lineage intestinal microbes, such as the Actinobacteria Collinsella, has been associated with autoimmune diseases in humans and may be important in dogs as well [10, 17, 41]. In our dog population, we identified one genera, Collinsella, in the Phyla Actinobacteria. We found that a significantly higher abundance of Actinobacteria (Table 2) in the Great Danes with GDV and at least one risk allele (Table A in S1 Tables, Tables A and B in S2 Tables, S1 Fig). An increase in Actinobacteria was also noted in mixed breed dogs by Allenspach et al in cases of chronic enteropathies [66]. The Actinobacteria Collinsella, has been linked autoimmune diseases in humans and humanized mice [28]. Similar to our GDV dogs (Table A in S1 Tables, Tables A and B in S2 Tables), humanized mice expressing a rheumatoid arthritis (RA) susceptible HLA gene had in a dysbiotic microbiota which was significantly enriched in Actinobacteria and, specifically, Collinsella [28]. Both mouse and cell line models showed stimulation of autoimmune response when exposed to Collinsella compared to *E*. *coli* controls. Collinsella appears to play a role in enhancing low level systemic inflammation as stimulated through the Th-17 regulatory network of cytokines. Exposure of Caco-2 cells to Collinsella resulted in increased permeability accompanied by decreased levels of zonulin, increased expression of IL-17A and inflammatory pathways activated through NFkB.

Recent studies have shown that mutations in microbial sensing genes, such as TLR5, can influence cross-talk between the host and the microbiota [13, 67]. In dogs with at least one risk allele of TLR5 and GVD, we found a significant decrease in alpha diversity coupled with the significant changes in bacterial community structure at the phyla which were reflected in the genera level. Actinobacteria (Collinsella by default) were significantly higher in dogs with one risk allele for TLR5). Strategies that influence the surveillance of the microbiome include mutation of the TLR5 genes and protein modification of flagellin by bacteria to make it unrecognizable by the TLR5 protein [26, 27, 39, 67]. Mutations in these genes are associated with dysbiotic microbial communities exhibiting decreased alpha diversity and when the altered microbiome is transferred to germ-free hosts, the communities are capable of inducing the disease state in a new host [27]. Other studies have shown a reduction in alpha diversity in TLR5 deficient and TLR5 mutant mice [26, 27, 67]. Studies showed that mice deficient in TLR5 were ineffective at controlling levels of the gamma- Proteobacteria, *E*. *coli* [37]. The gamma Proteobacteria were able to evade immune surveillance and proliferate due to reduced levels of anti-flagellin antibodies produced in mutant TLR5 hosts. In contrast, the bacteria may alter flagellin to evade host surveillance by TLR5. In human intestinal epithelial cells (IECs) exposed to flagellated Lactobacillus and isolated Lactobacillus flagellin, there was a robust inflammatory response by increased secretion of IL8 through the NfkB pathway [39]. Characterization of the Lactobacillus flagellin showed a protein modification that affected host recognition by TLR5. These data suggest that multiple strategies that impair the host’s ability to detect bacteria may lead to an altered microbiome as seen in GVD.

Adaptive immune genes play a role in controlling the composition of the microbiome in both humans and mice [30, 32, 68]. The MHC includes HLA class I and class II genes in humans, and similarly, DLA class I (DLA88) and DLA Class II (DRB1) genes in dogs. These two classes are responsible for antigen presentation and pathogen clearance and, thus, are among the most polymorphic (within species) and conserved (between species). In our study, we found that there were significant associations of bacterial phyla with mutations in Class II genes in GDV dogs compared to the controls. Firmicutes were significantly higher in GDV dogs (Table 2) and one risk allele for DLA Class II. In humans, mutations in Class II genes have been associated with autoimmune diseases such as celiac disease and rheumatoid arthritis [69–75]. Similar patterns have been observed in a humanized mouse model of rheumatoid arthritis [38]. The class II HLA-DRB1*04 allele was associated with increases in Clostridium (Phylum Firmicutes) with concomitant decreases in members of the Porphyromonadaceae family in the Phylum Bacteroidetes [38]. In contrast, Bifidobacteria, in the phylum Actinobacteria, were associated with increased Th17 profiles and loss of age- and sex-dependent microbiome [38]. In humans, mutation in HLA-DR genes have been associated rheumatoid arthritis, and increases in *P*. *copri* and reduced numbers of Bacteroides [76]. Mutations in the HLA genes have been associated with celiac disease and higher numbers of Firmicutes and Proteobacteria, and lower numbers of Actinobacteria were found in infants that carried the HLA mutation [68].

Our cross-sectional study has uncovered several associations that suggest underlying microbial and genetic associations in this multifactorial disease. One of the strengths of this study is we were able to associate host genetic mutations to the carriage of specific bacterial groups to a disease outcome. This is due to the study design which let us analyze host genotype and the microbiome. There are some weaknesses in the study. The timing of the sample collection relative to event may affect the microbiome. Since we collected stool sample after the GDV event, we cannot be sure that the data reflects the predisposing profile of the microbiome as opposed to the lingering effects of the GDV event, or the subsequent surgery and antibiotic treatment. While we tried to minimize the effects of these variables, by restricting sample collection to at least 3 months after any gastric distress, surgery or antibiotics, they cannot be ruled out. The best way to control for this caveat would be to sample a large pool of healthy dogs, and wait to see which ones experienced GDV. Such a longitudinal approach can be prohibitively expensive and time consuming. The sample size is small for a cross-sectional study and some trends were attenuated upon correction for multiple comparisons. Nonetheless, we found significant differences in the microbiome of dogs that had experienced GDV. The microbiome data are compositional data which has methodological artifacts associated with the standard normalization approach. Although we used a metric to account for this, it is an active area of computational research [58]. Despite this limitation, strong data signals are apparent in any normalization approach used. To understand the physiological interaction between the dog and the microbiome that leads to GDV, understanding the functional metabolism of the microbiome is important. In this study, we used 16S rRNA genes to measure composition. In the future, metagenomic and metatranscriptomic approaches coupled with isolation of novel canine bacteria using novel community culturing techniques would broaden our understanding of the microbial mechanisms involved in GDV. This will lay the groundwork to develop a prevention strategy that targets the microbial mechanisms involved in GDV. However, the stool samples from the dogs were taken subsequent to the GDV event and cannot establish causality or rule out effects of the disease or treatment. Future studies that follow a high-risk breed prospectively over time would establish the role of the dynamic microbiome in GDV and potentially lead to preventive measures therapeutics which may alter the trajectory to GDV.

## Conclusions

Multifactorial diseases are dependent on interactions between genetic and environmental factors. While genetic factors may impact both adaptive and innate immunity and may provide the highest risk factor for these diseases, environmental factors also appeared to be important. Here, we addressed an alternative hypothesis for a genetic link to GDV, namely, that variations in the genes of the immune system act through modulation of the gut microbiome and predispose dogs to this condition. Differences in the microbiome composition suggest that microbial mechanisms may underpin the etiology of GDV, modulating the phenotypes associated with the genetic factors that predispose the large breed dogs to GDV. While the cross-sectional nature of our study design only allows us to establish associations between genetics, the microbiome, and risk of GDV, it lays the foundation for future studies to establish the causality and develop a therapeutic approach to reduce risk of GDV in large breed dogs.

## Supporting information

S1 Fig ROC Curve for prediction of GVD using at least one risk allele, Actinobacteria, and temperament. (PDF)

S1 Tables **A)** Genera (%) that were significantly different in dogs with and without GDV. **B)** Data for the ROC curves with the false positive fraction (FPF), true positive fraction (TPF) and lower and upper confidence intervals (CI).
(DOCX)

S2 Tables **A)** Backward Stepwise Logistic Regression Model Estimating Effects of At Least One Risk Allele, Actinobacteria, and Temperament of the Risk of GDV (n = 65) and **B)** Goodness of Fit measures.
(DOCX)
