# Supplementary material for: The canine gut microbiome is associated with higher risk of gastric dilatation-volvulus and high risk genetic variants of the immune system
Source: PLoS One. 2018 Jun 11;13(6):e0197686. doi: 10.1371/journal.pone.0197686 (PMC5995382; doi:10.1371/journal.pone.0197686)

S1 Fig. ROC Curve for prediction of GVD using at least one risk allele, Actinobacteria, and temperament. (AUC=0.864)

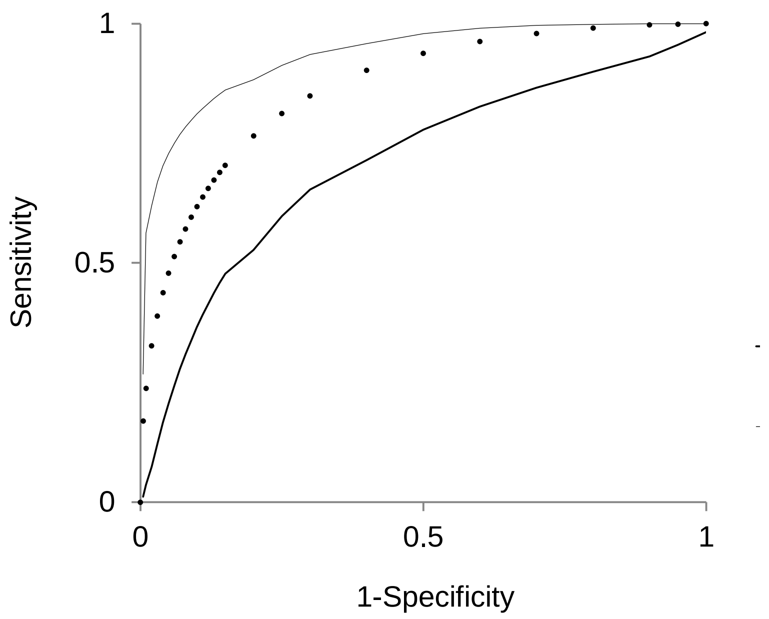

Supplement: S1 Fig — (PDF) [file pone.0197686.s001.pdf]
