# Supplementary material for: The canine gut microbiome is associated with higher risk of gastric dilatation-volvulus and high risk genetic variants of the immune system
Source: PLoS One. 2018 Jun 11;13(6):e0197686. doi: 10.1371/journal.pone.0197686 (PMC5995382; doi:10.1371/journal.pone.0197686)
Supplement: S1 Tables — A) Genera (%) that were significantly different in dogs with and without GDV. B) Data for the ROC curves with the false positive fraction (FPF), true positive fraction (TPF) and lower and upper confidence intervals (CI). (DOCX) [file pone.0197686.s002.docx]

| **S1 Tables.**   1. Genera (%) that are significantly different in dogs with and without GDV | | | | |
| --- | --- | --- | --- | --- |
| **Controls*** n=38 | | **GDV*** n=37 | ***P***** | ***P****** |
| Bacteria;Actinobacteria; Coriobacteriia;  Coriobacteriales; Coriobacteriaceae;  **Collinsella** | 0.14 (0.34) | 0.49 (0.65) | 0.001 | 0.063 |
| Bacteria;Bacteroidetes; Bacteroidia;  Bacteroidales; Prevotellaceae; **Prevotella** | 45.0 (28.0) | 29.7 (23.3) | 0.018 | 0.118 |
| Bacteria;Firmicutes; Bacilli; Lactobacillales;  Lactobacillaceae; **Lactobacillus** | 0.22 (0.93) | 3.83 (12.94) | 0.007 | 0.087 |
| Bacteria;Firmicutes; Clostridia; **Clostridiales**; Other;Other | 0.28 (0.58) | 0.87 (2.15) | 0.006 | 0.087 |
| Bacteria;Firmicutes; Clostridia; Clostridiales;  **Peptostreptococcaceae**;**Other** | 0.07 (0.20) | 0.18 (0.41) | 0.047 | 0.232 |
| Bacteria;Firmicutes; Clostridia; Clostridiales;  **Peptostreptococcaceae Incertae_Sedis** | 0.12 (0.35) | 0.77 (0.21) | 0.016 | 0.118 |
| Bacteria;**Proteobacteria**;Other;Other;Other;Other | 0.00 (0.00) | 0.04 (0.08) | 0.007 | 0.100 |
| Bacteria;Proteobacteria; Epsilonproteobacteria; Campylobacterales; **Helicobacteraceae**; _Helicobacter | 0.1 (0.24) | 0.05 (0.18) | 0.023 | 0.134 |
| Bacteria;Proteobacteria; Gammaproteobacteria;_  _Enterobacteriales; Enterobacteriaceae;  **Esc-Shigella** | 0.05 (0.23) | 0.33 (0.67) | 0.005 | 0.087 |
| Bacteria;Proteobacteria; **Gammaproteobacteria**; Other;Other;Other | 0.98 (4.23) | 3.30 (7.5) | 0.011 | 0.100 |

***** Mean (SD)

** Non parametric t-test (Kruskal Walis) uncorrected for multiple comparisons

******* Non parametric t-test (Kruskal Walis) Benjamini Hochberg correction for multiple comparisons

| **b**) Data for the ROC curves with the false positive fraction (FPF), true positive fraction (TPF) and lower and upper confidence intervals (CI). | | | |
| --- | --- | --- | --- |
| FPF | TPF | Lower CI | Upper CI |
| 0 | 0 | 0 | 0 |
| 0.005 | 0.1699 | 0.0229 | 0.5348 |
| 0.01 | 0.2379 | 0.0491 | 0.5899 |
| 0.02 | 0.3267 | 0.0994 | 0.6504 |
| 0.03 | 0.3891 | 0.1455 | 0.6888 |
| 0.04 | 0.4381 | 0.1877 | 0.7174 |
| 0.05 | 0.4788 | 0.2262 | 0.7405 |
| 0.06 | 0.5136 | 0.2617 | 0.7599 |
| 0.07 | 0.544 | 0.2944 | 0.7768 |
| 0.08 | 0.571 | 0.3247 | 0.7917 |
| 0.09 | 0.5953 | 0.3529 | 0.8051 |
| 0.1 | 0.6174 | 0.3791 | 0.8173 |
| 0.11 | 0.6376 | 0.4035 | 0.8285 |
| 0.12 | 0.6561 | 0.4264 | 0.8387 |
| 0.13 | 0.6733 | 0.4478 | 0.8483 |
| 0.14 | 0.6892 | 0.468 | 0.8571 |
| 0.15 | 0.7041 | 0.4869 | 0.8654 |
| 0.2 | 0.7657 | 0.567 | 0.8999 |
| 0.25 | 0.8124 | 0.6287 | 0.9258 |
| 0.3 | 0.849 | 0.6778 | 0.9455 |
| 0.4 | 0.9023 | 0.7517 | 0.9719 |
| 0.5 | 0.9381 | 0.8056 | 0.9867 |
| 0.6 | 0.9628 | 0.8481 | 0.9945 |
| 0.7 | 0.9797 | 0.8838 | 0.9981 |
| 0.8 | 0.9907 | 0.9159 | 0.9996 |
| 0.9 | 0.9973 | 0.9473 | 1 |
| 0.95 | 0.9991 | 0.9649 | 1 |
| 1 | 1 | 1 | 1 |
