# Supplementary material for: The canine gut microbiome is associated with higher risk of gastric dilatation-volvulus and high risk genetic variants of the immune system
Source: PLoS One. 2018 Jun 11;13(6):e0197686. doi: 10.1371/journal.pone.0197686 (PMC5995382; doi:10.1371/journal.pone.0197686)
Supplement: S2 Tables — A) Backward Stepwise Logistic Regression Model Estimating Effects of At Least One Risk Allele, Actinobacteria, and Temperament of the Risk of GDV (n = 65) and B) Goodness of Fit measures. (DOCX) [file pone.0197686.s003.docx]

| **S2 Tables**  **a)** Backward Stepwise Logistic Regression Model Estimating Effects of At Least One Risk Allele, Actinobacteria, and Temperament of the Risk of GDV (n = 65) and 2 **b)** Goodness of Fit measures. | | | | | | | |
| --- | --- | --- | --- | --- | --- | --- | --- |
| **a)** |  |  |  |  |  |  |  |
| Source | Value | Standard error | Wald Chi- Square | Pr > Chi² | Odds ratio | Odds ratio Lower  bound (95%) | Odds ratio Upper  bound (95%) |
| Intercept | -1.66 | 4.08 | 0.17 | 0.68 | . |  |  |
| Bacteria; Actinobacteria | 0.31 | 0.15 | 4.05 | 0.04 | 1.37 | 1.01 | 1.85 |
| Temperament-Nervous | 2.02 | 0.86 | 5.50 | 0.02 | 7.55 | 1.39 | 40.89 |
| One Risk allele-1 | 2.52 | 0.75 | 11.12 | 0.00 | 12.39 | 2.82 | 54.41 |

| **b)** | |
| --- | --- |
| Statistic | Independent |
| Observations | 65 |
| Sum of weights | 65.00 |
| DF | 64 |
| -2 Log(Likelihood) | 90.09 |
| R²(McFadden) | 0.00 |
| AIC | 92.09 |
| SBC | 94.26 |
| Iterations | 0 |
